# Supplementary material for: Modelling transmission thresholds and hypoendemic stability for onchocerciasis elimination
Source: PLoS Comput Biol. 2025 Apr 21;21(4):e1013026. doi: 10.1371/journal.pcbi.1013026 (PMC12052207; doi:10.1371/journal.pcbi.1013026)
Supplement: S1 Text — Text A. Brief description of EPIONCHO-IBM. Table A. Density dependence parameters determining parasite establishment within humans as a function of the annual transmission potential (ATP, no. L3/person/year) for different values of parameter kE, of the gamma distribution describing inter-individual exposure heterogeneity. Fig A. Schematic representation of deterministic versus stochastic projections for low infection prevalence. Fig B. Initialising EPIONCHO-IBM with an endemic equilibrium. Fig C. Model-derived and fitted proportion of EPIONCHO-IBM runs persisting over time. Text B. Modelling for policy: PRIME-NTD. Table B. Policy-Relevant Items for Reporting Models in Epidemiology of Neglected Tropical Diseases (PRIME-NTD) summary table. Fig D. EPIONCHO-IBM predicted distribution of microfilarial (mf) prevalence over simulation time. Fig E. Influence of human population size (N) on model run persistence distributions. Fig F. Influence of inter-individual heterogeneity parameter, kE, on persistence of transmission. (DOCX) [file pcbi.1013026.s001.docx]

# **Supporting Information S1 Text**

# **Modelling transmission thresholds and hypoendemic stability for onchocerciasis elimination**

Jacob N. Stapley^1, *^, Jonathan I.D. Hamley^1,2,3^, Maria-Gloria Basáñez^1, †^, Martin Walker^1,4, †^

^1^ MRC Centre for Global Infectious Disease Analysis and London Centre for Neglected Tropical Disease Research, Department of Infectious Disease Epidemiology, School of Public Health, Imperial College London, London, UK

^2^ Department of Visceral Surgery and Medicine, Inselspital, Bern University Hospital, University of Bern, Switzerland

^3^ Multidisciplinary Center for Infectious Diseases, University of Bern, Switzerland

^4^ Department of Pathobiology and Population Sciences, Royal Veterinary College, Hatfield, UK

*Corresponding Author

Email: [j.stapley20@imperial.ac.uk](mailto:j.stapley20@imperial.ac.uk)

^†^Joint senior authors

**Text A. Brief description of EPIONCHO-IBM**

EPIONCHO-IBM is a stochastic, individual-based model [1] developed from its deterministic, population-based (EPIONCHO) predecessors [2–4]. The model tracks, in a closed population of *N* individuals, the number of adult (male and female) *O. volvulus* worms in human hosts, the number of microfilariae in their skin, and the number of infective, L3 larvae in blackfly vectors. Parasite population abundance is regulated in humans and blackflies by density-dependent processes operating on the establishment of incoming worms within humans; establishment of L3 larvae within vectors, and vector survival [1,3,4]. Excess mortality of humans as a function of microfilarial load [5] has not yet been included. For sub-Saharan Africa settings, the model has been parameterised for savannah *Onchocerca volvulus–Simulium damnosum* sensu lato (s.l.) [1–4]. The baseline (pre-control) endemicity (given by microfilarial, mf, prevalence in those aged ≥ 5 years) is determined by the annual biting rate (ABR, no. bites/person/year) [1,4].

Individuals within the model are differentially exposed to blackfly bites depending on their age and sex [2] as well as on their individual-specific exposure, $E_{(i)}$ [1]. This individual exposure factor is assigned at birth and drawn from a gamma distribution,

| $E_{(i)}\sim G(k_{E},k_{E})$ | (S1) |
| --- | --- |

where $k_{E}$ is equal to the shape and rate parameters, such that the mean exposure in the population is unity, i.e., blackfly bites are distributed among hosts with an average exposure given by the annual biting rate (ABR, no. bites/person/year) [1]. Estimated values of $k_{E}$ (ranging from 0.2 to 0.4) are accompanied by specific sets of density dependence parameters such that lower values of $k_{E}$ (indicating stronger exposure heterogeneity among hosts) correspond to stronger density dependence in parasite establishment within humans as a function of the annual transmission potential (ATP, no. L3/person/year) and vice versa [1]. Exposure heterogeneity and density dependence parameters were estimated by fitting EPIONCHO-IBM to data on mf prevalence and mf load as a function of ABR [1] (Table A). Therefore, for a given ABR and $k_{E}$, EPIONCHO-IBM generates the adult worm burden and mf load of each individual host in the population, also allowing for calculation of mf prevalence in the population (for any age group, e.g., those aged ≥5 years).

The mortality rates of both adult worms [6,7] and microfilariae [8] are assumed to increase as a function of parasite age, according to a Weibull distribution of survival times [1]. Newly established adult female *O. volvulus*, are initially non-fertile, and progress to become fertile. Female worms only produce microfilariae when in the fertile state and in the presence of at least one co-infecting male worm (assuming complete polygamy of male worms such that one male can mate with all females within the same human host). Female worms undergo 3–4 re-insemination cycles per year [9,10] and have maximum fecundity during the first 5 years of age followed by a linear reduction in fecundity, which becomes zero for worms older than 20 years [1]. The change in the density of microfilariae per mg of skin is calculated deterministically [1].

The vector transmission cycle is also modelled deterministically. An individual’s contribution to the larval burden in the vector population is calculated as a function of their microfilarial load [1,2]. This contribution depends on the biting rate per fly on humans, and the individual’s specific relative exposure to blackfly bites. Much like the establishment of L3 larvae in the human host, the establishment of microfilariae in the simuliid vector is determined by a constraining density-dependent function, such that the proportion of microfilariae developing into infective L3 larvae within the blackfly vector declines with increasing number of microfilariae ingested [11]. Additionally, this proportion is affected by the probability that a blackfly survives the extrinsic incubation period (EIP), the time it takes for ingested microfilariae to develop into L3 larvae, which represents a substantial proportion of the blackfly’s lifespan [11]. The EIP is incorporated by modelling explicitly the L1, L2 and L3 stages of development within the blackfly vector [4] whilst also accounting for a delay before L1 can start transitioning to the L2 stage [1], as suggested by experimental blackfly infection data [11]. A complete description of EPIONCHO-IBM is presented in the Supplementary File of Hamley et al. [1], and the R code can be found at: <https://github.com/mrc-ide/EPIONCHO.IBM>.

**Table A.** Density dependence parameters determining parasite establishment within humans as a function of the annual transmission potential (ATP, no. L3/person/year)) for different values of parameter $k_{E}$ of the gamma distribution describing inter-individual exposure heterogeneity.

| Shape and rate parameter of gamma distribution | Density dependence parameters for parasite establishment within humans | | |
| --- | --- | --- | --- |
| $k_{E}$ | $\delta_{H_{0}}$ | $\delta_{H_{\infty}}$ | $c_{H}$ |
| 0.2 | 0.385 | 0.003 | 0.008 |
| 0.3 | 0.186 | 0.003 | 0.005 |
| 0.4 | 0.118 | 0.002 | 0.004 |

$\delta_{H_{0}}$ is the proportion of L3 larvae developing to adult worms within the human host, per bite, when ATP tends to 0; $\delta_{H_{\infty}}$ is the proportion of L3 larvae developing to adult worms within the human host, per bite, when ATP is very large, and $c_{H}$ is the severity of transmission intensity-dependent parasite establishment within humans [1–4].


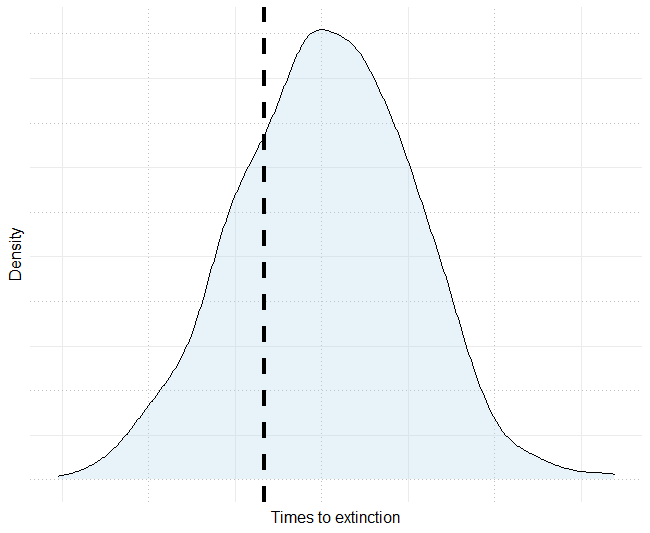

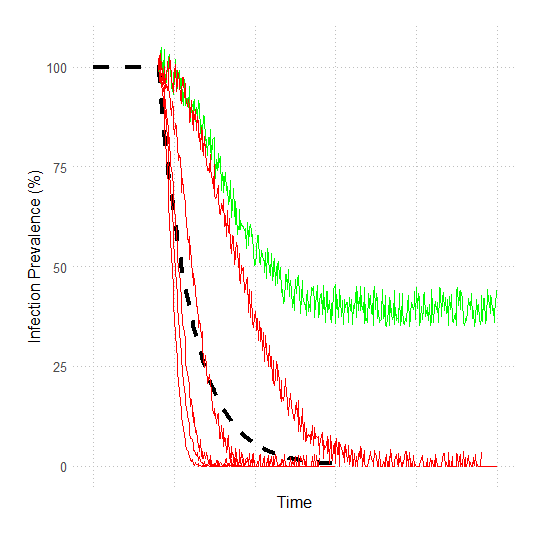


**Fig A. Schematic representation of deterministic versus stochastic projections for low infection prevalence.** When run from a pre-established equilibrium using a fixed parameter set below the threshold for endemic persistence, a deterministic model predicts a single decay in infection, since the annual biting rate (ABR) is below the transmission threshold (i.e., *R*_0_ is <1) and, therefore, infection cannot persist. A stochastic model inherently generates uncertainty, predicting a distribution of outcomes from the same input parameters. Panel A, dashed line, shows a hypothetical deterministic infection model in contrast to the runs for its stochastic analogue, which can predict local extinction after different time periods (red lines) or infection persistence (green line) under identical initial transmission conditions. This is reflected in the distribution of times to reach 0% infection prevalence (Panel B), with a stochastic model giving a distribution of potential outcomes (shaded area), in contrast to the fixed deterministic outcome (dashed line).

**A**

**B**

**Fig B. Initialising EPIONCHO-IBM with an endemic equilibrium.** Three hundred model repeats for an annual biting rate, ABR = 190, human population size, *N* = 400, and inter-individual heterogeneity parameter, *k_E_* = 0.3 were simulated for 500 years, starting from four equilibria generated by different ABRs: 40,000, 10,000, 1,000 and 250 bites/person/year. Even with variation across orders of magnitude, microfilarial (mf) prevalence dynamics (coloured lines) are nearly indistinguishable after 80 years of simulation, with the final proportion of simulations persisting (i.e., > 0% mf prevalence) after 500 years (coloured markers, right hand y-axis) being contained within a narrow range. Consequently, an ABR of 1,000 bites/person/year was used to generate initial conditions for all subsequent simulations presented in the Main Text and Figs C–F.


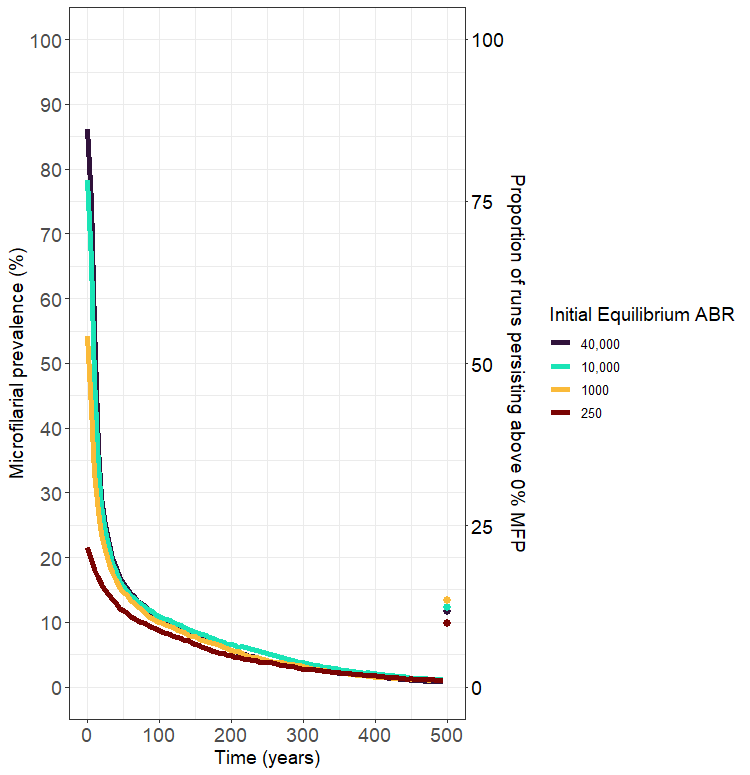


**Text B. Modelling for policy: PRIME-NTD**

For the analyses presented, we adhered to the Five Principles of the Neglected Tropical Diseases (NTD) Modelling Consortium for good practice in policy-relevant NTD modelling [12]. Table B briefly describes the five tenets, how they were fulfilled, and where in the Main Text and/or Supplementary Information they can be found.

**Table B.** Policy-Relevant Items for Reporting Models in Epidemiology of Neglected Tropical Diseases (PRIME-NTD) summary table [12].

| **Principle** | **What has been done to satisfy the principle?** | **Where in the manuscript is this described?** |
| --- | --- | --- |
| **Stakeholder engagement** | Discussions with modelling and policy-focused collaborators | Author list, Acknowledgements section |
| **Complete model documentation** | References to the full description of EPIONCHO-IBM are provided. An Open Access link to the code has been given | Methods section, S1 Text and Data availability statement |
| **Complete description of data used** | We compare our findings with published data adequately referenced | Main text, Reference list of Main Text |
| **Communicating uncertainty** | Sensitivity analyses were conducted to investigate the influence of varying population size and inter-individual exposure heterogeneity. The ABR was varied to generate the initial conditions during the model burn-in period as well as determining which survivorship model best fitted EPIONCHO-IBM outputs. | Methods and Results sections. Figures and figure legends. Figs A-F in S1 Text. |
| **Testable model outcomes** | Findings were compared to epidemiological data. Model projections of persistent hypoendemicity could be compared to future longitudinal studies. The effect of commencing treatment in hypoendemic foci could be tested. | Results and Discussion sections |

**Fig C. Model-derived and fitted proportion of EPIONCHO-IBM runs persisting over time.** Weibull models (coloured lines) were fitted to the proportion of persistent runs (black markers) from the final time step which gave 100% model run persistence until the end of the 500-year simulation period to generate the fade-out rates presented in Fig. 2 of the Main Text. The simulation time necessary to reach the timepoint from which the Weibull models were fitted increased alongside annual biting rate (ABR) because for higher ABRs runs persisted for longer. Eight ABRs (210, 200, 190, 180, 160, 140, 120 and 100 bites/person/year; panels A-H) were selected to give values near the maximum of the threshold biting rate (TBR) range down to an ABR below the TBR range. Model outputs and fits are shown for a modelled population of *N* = 400 humans and inter-individual exposure heterogeneity parameter, *k_E_* = 0.3.


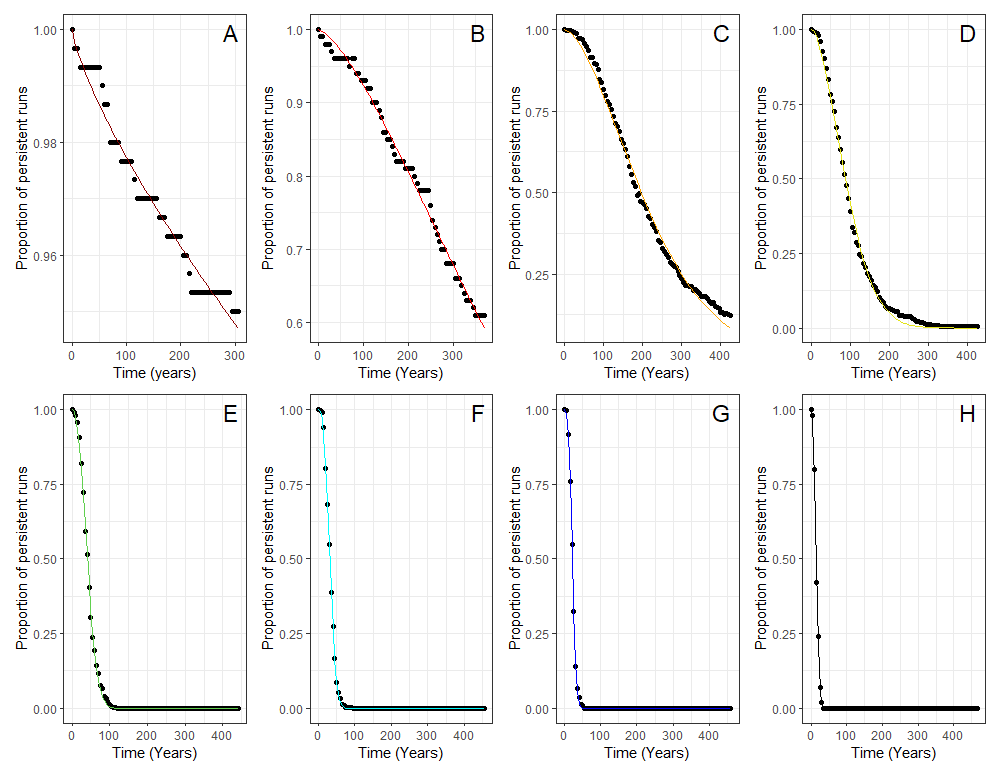

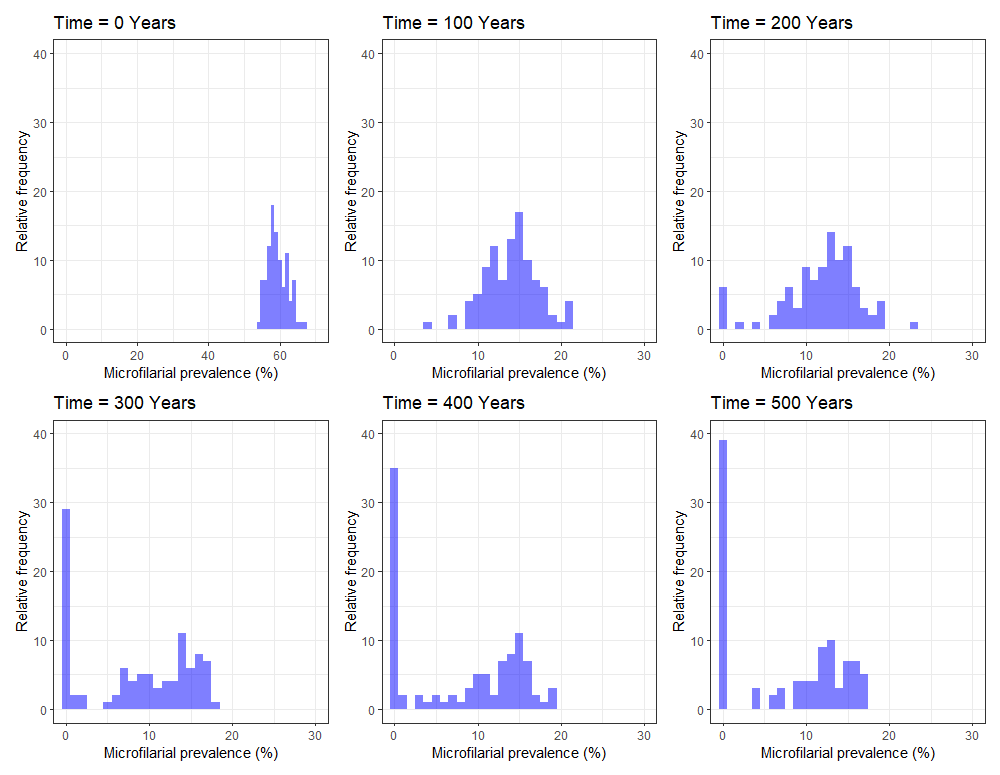


**F**

**E**

**D**

**C**

**B**

**A**

**Fig D. EPIONCHO-IBM predicted distribution of microfilarial (mf) prevalence over simulation time.** The blue bars show the relative frequency of a simulated mf prevalence generated by 300 model repeat simulations at 100-year intervals (0–500 years; Panels A–F) for a modelled population of *N* = 400 humans, inter-individual exposure heterogeneity parameter, *k_E_* = 0.3, and annual biting rate of 1,000 bites/person/year decreasing to a threshold biting rate of 200 bites/person/year after the first time step.


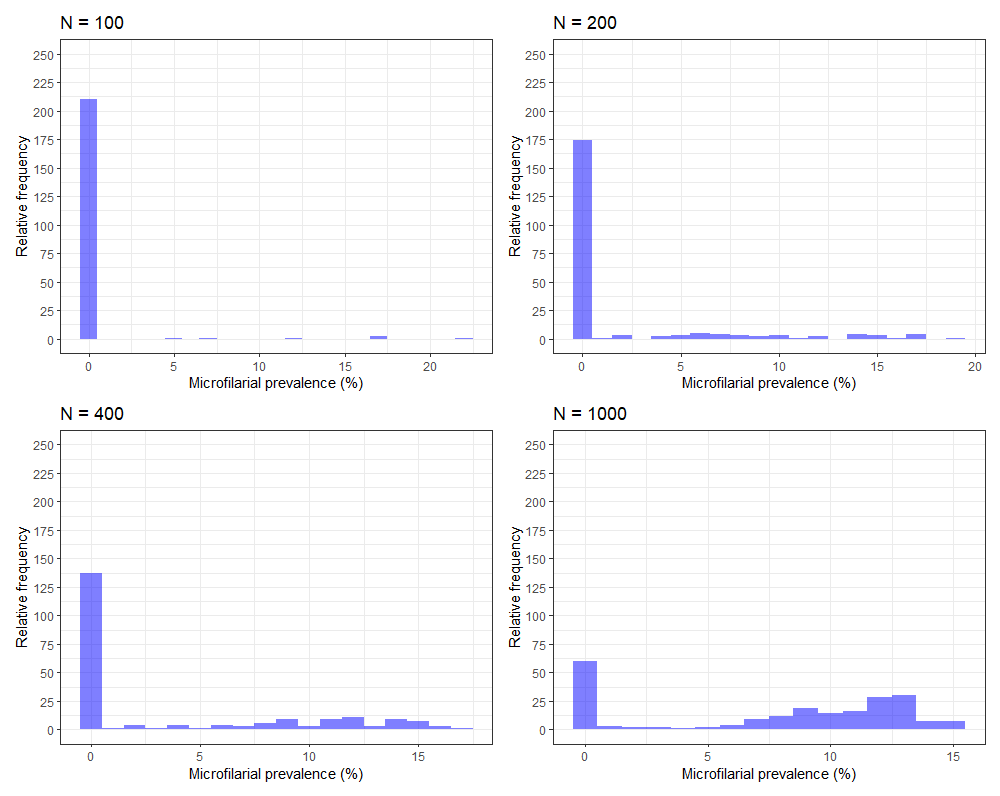


**Fig E. Influence of human population size (*N*) on model run persistence distributions.** The blue bars show the relative frequency of a simulated mf prevalence generated by 300 simulations of EPIONCHO-IBM after 500 years with a fixed biting rate of 195 bites/person/year and inter-individual exposure heterogeneity parameter *k_E_* = 0.3. As population size increases from *N* = 100, 200, 400, to 1000 (Panels A–D, respectively) a greater proportion of runs persist.

**A**

**B**

**C**

**D**


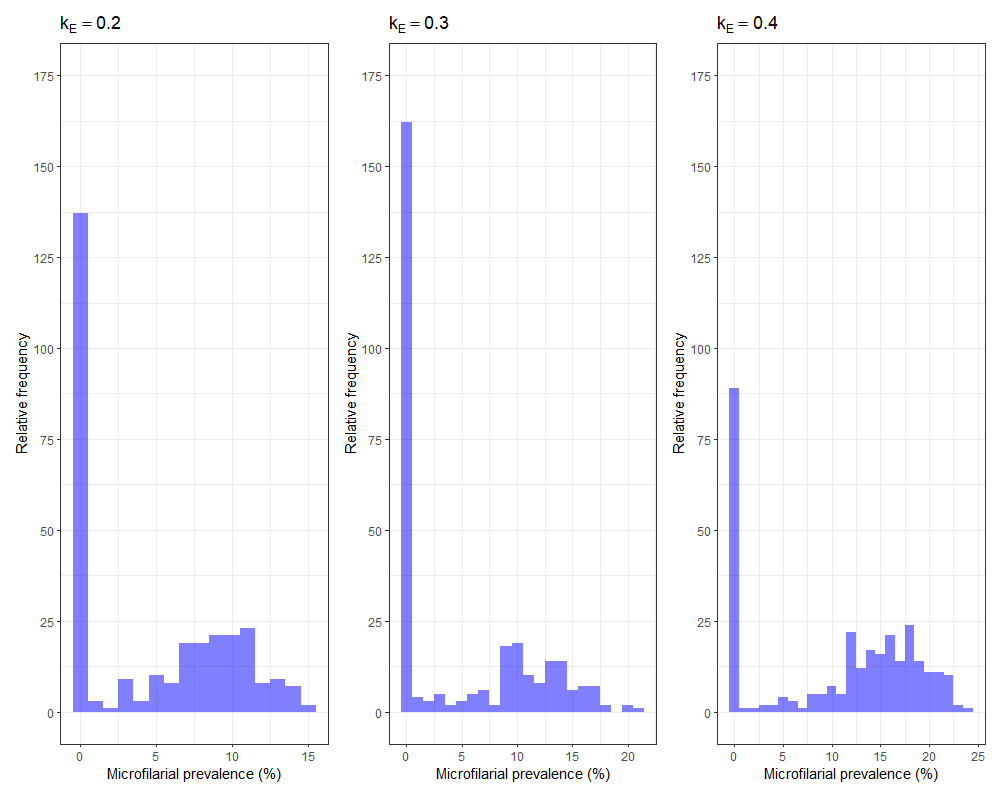


**Fig F. Influence of inter-individual heterogeneity parameter, *k_E_*, on persistence of transmission.** The blue bars show the relative frequency of simulated microfilarial (mf) prevalence generated by 300 repeat simulations of EPIONCHO-IBM after 500 years at a fixed population size of *N* = 400 for values of parameter *k_E_* = 0.2, 0.3, 0.4 (Panels A–C, respectively). Mf prevalence was generated using annual biting rates (ABR) (80, 197 and 360 bites/person/year, respectively) which gave comparable proportions of persisting runs. A lower *k_E_* value (indicating stronger exposure heterogeneity) stabilises transmission at lower ABRs.

**A**

**B**

**C**

**Supplementary References**

1. Hamley JID, Milton P, Walker M, Basáñez MG. Modelling exposure heterogeneity and density dependence in onchocerciasis using a novel individual-based transmission model, EPIONCHO-IBM: implications for elimination and data needs. PLoS Negl Trop Dis. 2019; 13: e0007557. <https://doi.org/10.1371/journal.pntd.0007557>.
2. Filipe JAN, Boussinesq M, Renz A, Collins RC, Vivas-Martinez S, Grillet ME, Little MP, Basáñez MG. Human infection patterns and heterogeneous exposure in river blindness. Proc Natl Acad Sci U S A. 2005; 102: 15265–70. <https://doi.org/10.1073/pnas.0502659102>.
3. Basáñez MG, Walker M, Turner HC, Coffeng LE, de Vlas SJ, Stolk WA. River blindness: mathematical models for control and elimination. Adv. Parasitol. 2016; 94: 247–341. <https://doi.org/10.1016/bs.apar.2016.08.003>.
4. Walker M, Stolk WA, Dixon MA, Bottomley C, Diawara L, Traoré MO, de Vlas SJ, Basáñez MG. Modelling the elimination of river blindness using long-term epidemiological and programmatic data from Mali and Senegal. Epidemics. 2017; 18: 4–15. <https://doi.org/10.1016/j.epidem.2017.02.005>.
5. Walker M, Little MP, Wagner KS, Soumbey-Alley EW, Boatin BA, Basáñez MG. Density-dependent mortality of the human host in onchocerciasis: relationships between microfilarial load and excess mortality. PLoS Negl Trop Dis. 2012; 6 :e1578. <https://doi.org/10.1371/journal.pntd.0001578>.
6. Plaisier AP, van Oortmarssen GJ, Remme J, Habbema JDF. The reproductive lifespan of *Onchocerca volvulus* in West African savanna. Acta Trop. 1991; 48: 271–284. <https://doi.org/10.1016/0001-706x(91)90015-c>.
7. Karam M, Schulz-Key H, Remme J. Population dynamics of *Onchocerca volvulus* after 7 to 8 years of vector control in West Africa. Acta Trop. 1987; 44: 445–57.
8. Duke BOL. The effects of drugs on *Onchocerca volvulus* I. Methods of assessment, population dynamics of the parasite and the effects of diethylcarbamazine. Bull World Health Organ. 1968; 39: 137–146.
9. Schulz-Key H, Karam M. Periodic reproduction of *Onchocerca volvulus*. Parasitol Today. 1986; 2: 284–86. <https://doi.org/10.1016/0169-4758(86)90138-9>.
10. Schulz-Key H. Observations on the reproductive biology of *Onchocerca volvulus*. Acta Leiden. 1990; 59: 27–44.
11. Basáñez MG, Churcher TS, Grillet ME. *Onchocerca–Simulium* interactions and the population and evolutionary biology of *Onchocerca volvulus*. Adv Parasitol. 2009; 68: 263–313. <https://doi.org/10.1016/S0065-308X(08)00611-8>.
12. Behrend MR, Basáñez MG, Hamley JID, Porco TC, Stolk WA, Walker M, *et al.* Modelling for policy: the five principles of the Neglected Tropical Diseases Modelling Consortium. PLoS Negl Trop Dis. 2020; 14: e0008033. <https://doi.org/10.1371/journal.pntd.0008033>.
