# Supplementary material for: Modelling transmission thresholds and hypoendemic stability for onchocerciasis elimination
Source: PLoS Comput Biol. 2025 Apr 21;21(4):e1013026. doi: 10.1371/journal.pcbi.1013026 (PMC12052207; doi:10.1371/journal.pcbi.1013026)
Supplement: Attachment — Submitted filename: Response_to_Reviewers_13.03.25.docx [file pcbi.1013026.s003.docx]

**REPLIES TO REFEREEES**

**PCOMPBIOL-D-24-01739**

**Reviewers' comments:**

**Reviewer #1:**

I have had the chance to review the manuscript titled “Modelling transmission thresholds and hypoendemic stability for onchocerciasis elimination”. This manuscript addresses a crucial issue in the elimination of onchocerciasis, one of the neglected tropical diseases, particularly the dynamics in hypoendemic areas that have not been prioritized for mass drug administration (MDA). It employs the EPIONCHO-IBM stochastic individual-based model to explore the thresholds for transmission and the stability of infection under varying population and exposure heterogeneity conditions. The findings emphasize the need for extending treatment to all areas with endogenous infections. This manuscript can be published in PLOS Computational after the authors have addressed the following concerns.

We thank Reviewer 1 for their appreciative comments on the importance of our work and for their recommendation of publication following revision.

**1.1:** The authors have not compared the model’s outputs with real-world data or previous empirical findings, thereby limiting the ability to gauge its practical applicability. The authors need to incorporate comparisons with field data or historical studies to validate the findings and enhance their credibility.

**Response to 1.1:**

We agree with the Reviewer that comparing our modelling outputs with empirical data is important. Due to the scarcity of data—particularly in low-transmission ‘hypoendemic’ settings which have traditionally been ignored by intervention programmes—a ‘formal validation’ of the model is not possible. However, we have enhanced our narrative with the comparison of our findings with some available studies on the stability of hypoendemic transmission. Particularly important is the study by Katabarwa *et al.* (2010), which provides empirical epidemiological evidence supporting our results.

In this study, individuals who were resident for at least 10 years within 10 proximate communities—experiencing similar ecological and entomological conditions—were surveyed 20 km from the nearest CDTI implementation zone (i.e., the communities had not received treatment and were deemed to be sufficiently far away from CDTI communities). The microfilarial prevalence in adults (aged >20 years) ranged from 0% to 11.8%, while prevalence in children (aged 3–10 years) ranged from 0% to 1.92%. These low levels of microfilarial prevalence among communities with similar transmission conditions aligns well with our model's predictions and suggest that these communities were experiencing a biting rate within our threshold biting rate (TBR) range. In fact, Figure 3 of the Katabarwa *et al*. study shows entomological data that permit a calculation of the ABR for 2008 and 2009, resulting in a mean of 178, well within our TBR range.

As shown in Figure 1 of our manuscript, some communities within the TBR range will experience spontaneous fade-out (i.e., 0% prevalence), while others will maintain persistent transmission. Similarly, in Figures 2 and 3, we illustrate that very low microfilarial prevalence (e.g., up to 12%) may persist for many decades without intervention, consistent with the low-prevalence infections reported in Katabarwa *et al.* (2010).

We have added text to the Discussion to emphasise the concordance of our findings with the epidemiological data from this study:

“Our findings are consistent with epidemiological observations at focus, national and regional levels in SSA. At focus level, a study in Cameroon, aimed at investigating whether autochthonous transmission occurs in hypoendemic areas, conducted a survey of 10 ivermectin-naïve communities situated along the river Mayo Douka in the Ngong Health District of Cameroon. The villages shared similar ecological and entomological conditions and there was no migration (participants were resident in their communities for at least 10 years prior to the study, and the villages were >20 km away from the nearest meso- ⁄ hyperendemic zone). The mf prevalence in adults (aged >20 years) ranged from 0% to 12%, and in children (aged 3–10 years) ranged from 0% to 2%. The average ABR over two years was 178 (within our TBR), with 0.016 L3 larvae per fly (indicative of active transmission). The authors concluded that Ngong is a hypoendemic focus with endogenous transmission in isolation from meso- and hyperendemic areas [13].”

**1.2:** Can the authors enhance Hypoendemic stability findings by including case studies or field data?

**Response to 1.2:**

We agree with the Reviewer that emphasising that our modelling findings align with observed epidemiological data is very important. While the general scarcity of longitudinal data in low-transmission settings prevents a ‘formal validation’ of our model outputs, we have included text in the Discussion to compare of our findings with empirical epidemiological observations (see **Response to 1.1**).

As described in **Response to 1.1**, the data from Katabarwa *et al.* (2010) support the notion that very low microfilarial prevalence (in the absence of intervention or importation via migration) can occur in communities that experience biting rates within the TBR range we report, illustrating a focus-level example.

A national-level example is that of Gabon:

“At national level, a systematic review, covering studies across a 26-year period from 1992-2018 in Gabon, reported nationwide microfilarial prevalence fluctuating between 42% in 1994 and 8% in 2006 in the absence of any control measures [36]. In 2015, of 31 surveyed districts, only four exhibited meso- or hyperendemicity, while the majority (87%) indicated hypoendemicity according to mf prevalence [37]. Neither ivermectin MDA nor vector control against onchocerciasis have ever been implemented in Gabon, suggesting that hypoendemicity can persist over decades.”

Finally, our findings are also supported by regional-level data:

“At regional level, our results are further supported by pre-intervention epidemiological data collected under the Onchocerciasis Control Programme in West Africa (OCP). This dataset comprised baseline mf prevalence from 737 villages (before the initiation of control interventions) [22]. Among these, 172 villages (23%) were classified as hypoendemic, with mf prevalence ranging between ≥10% and <35%, indicating that hypoendemicity was not a rare phenomenon in West Africa, attesting to the occurrence of stable hypoendemic transmission. Notably, O’Hanlon *et al.* (2016) [22] categorised <10% microfilarial prevalence as ‘non-endemic/sporadic endemicity’, a stratification which aligns well with our modelling projections. In Fig. 3 of our study, we show that biting rates generating <10% prevalence fall below the threshold biting rate (TBR) range, making them susceptible to spontaneous fade-out, albeit this could take place over many decades.”

We have also included the following text on the Discussion:

“We see that (when *N* = 400 and *k_E_* = 0.3) an ABR of 210 generates 16% mf prevalence, the lowest prevalence which does not fade-out over the simulation period. Below 16%, fade-out occurs over time. This aligns with the endemicity stratifications used in O’Hanlon *et al.* [22], where <10% mf prevalence was deemed ‘non-endemic/sporadic endemicity.’ However, even below 10% mf prevalence (and below our estimated TBR range), the rate of fade-out of infection can be slow.”

The new references are:

O’Hanlon SJ, Slater HC, Cheke RA, Boatin BA, Coffeng LE, Pion SDS, *et al*. Model-based geostatistical mapping of the prevalence of *Onchocerca volvulus* in West Africa. PLoS Negl Trop Dis. 2016; 10: e0004328.

Eyang-Assengone E-R, Makouloutou-Nzassi P, Mbou-Boutambe C, Bangueboussa F, Atsame J, Boundenga L. Status of onchocerciasis elimination in Gabon and challenges: a systematic review. Microorganisms. 2023; 11: 1946.

**1.3:** This can also be extended to include discussion of the implications of potential discrepancies between model predictions and observed data.

**Response to 1.3:**

We acknowledge the Reviewer’s important point regarding potential discrepancies between our model predictions and observed epidemiological data, while also emphasising that a ‘formal validation’ of the model predictions with observed data is not possible (see **Response to 1.1** and **Response to 1.2**). Notwithstanding, and as discussed in the manuscript, a key feature of the model is the inclusion of density-dependent processes that can stabilise transmission at low prevalence. While these could potentially overestimate persistence compared to real-world observations, we emphasise the broad concordance between our findings and the epidemiological data outlined above. Models that omit parasite population regulation taking place within humans (e.g., ONCHOSIM) cannot model persistent transmission below ABR values of 8,000–10,000 (de Vos *et al*., 2021). We have added the following text to the Discussion:

“When calibrating EPIONCHO-IBM with the Gabon mf data for hypoendemic villages [37] (mean observed mf prevalence = 5.5%; mean modelled mf prevalence = 5%), the best-fit parameters were ABR = 73 and *k*_E_ = 0.2 [38], consistent with our results. This is in contrast with other models which are unable to generate low mf prevalence endemic stability at these low ABRs, in closed populations without a rate of infection importation from external sources [34,39], but see [40].”

The new references are:

de Vos AS, Stolk WA, Coffeng LE, de Vlas SJ. The impact of mass drug administration expansion to low onchocerciasis prevalence settings in case of connected villages. PLoS Negl Trop Dis. 2021; 15: e0009011.

de Vos AS, Stolk WA, de Vlas SJ, Coffeng LE. The effect of assortative mixing on stability of low helminth transmission levels and on the impact of mass drug administration: model explorations for onchocerciasis. PLoS Negl Trop Dis. 2018; 12: e0006624.

We have also revised the manuscript to discuss other relevant processes that will affect the stability of the model in low-transmission settings which were omitted from the original version of the manuscript. For example, EPIONCHO-IBM does not account for the effects of excess mortality associated with high microfilarial load (Little *et al.*, 2004; Walker *et al.*, 2012). Removal of individuals with high loads would increase the rate of fade-out. Further, the model assumes a closed population, meaning that migration is not incorporated into the model dynamics. In reality, migration from higher endemicity foci could assist in stabilising hypoendemic transmission (Nikièma *et al.*, 2018), potentially acting in the opposite direction to excess human mortality. Together, these competing processes illustrate the complex interplay between demographic and epidemiological factors that potentially contribute to hypoendemic stability and could alter the persistence of low prevalence.

We have added text to the Discussion to provide a more comprehensive exposition of processes that could affect our predictions on hypoendemic stability and persistence, emphasising the need for longitudinal epidemiological data for a more formal validation:

“Other factors not explored in this paper will also influence the TBR. The model does not include excess mortality associated with infection in the human population [4,5], which could, in principle, decrease stability and persistence by reducing the lifespan of individuals with high mf loads. However, we consider this effect to be of limited significance in the low-transmission settings considered. More pertinent, is the potential impact of imported infections via immigration which could enhance stability [51]. Here we have assumed a closed population, with stability mediated by (down-regulating) parasite density-dependent processes which enhance the efficiency of transmission at low endemicity. Very little is known on patterns of human and/or blackfly movement among rural endemic communities [39,52] and so it is currently impossible to disentangle the contributions of importation and density dependence on endemic stability. This topic warrants additional research, not only in terms of understanding the causes of low-level endemic persistence, but also critically for the risk of re-introduction of infection where elimination has been reached.”

The new references are:

Nikièma AS, Koala L, Post RJ, Paré AB, Kafando CM, Drabo F, *et al.* Onchocerciasis prevalence, human migration and risks for onchocerciasis elimination in the Upper Mouhoun, Nakambé and Nazinon river basins in Burkina Faso. Acta Trop. 2018; 185: 176–82.

Pindolia DK, Garcia AJ, Huang Z, Smith DL, Alegana VA, Noor AM, Snow RW, Tatem AJ. The demographics of human and malaria movement and migration patterns in East Africa. Malar J. 2013; 12: 397.

**1.4:** The manuscript emphasizes stochastic dynamics but does not sufficiently address the variability across different ecological and social contexts, such as co-endemicity with other diseases. The authors need to address the implications of the model’s findings in co-endemic areas, especially where *Loa loa* is prevalent. This when addressed can provide a clear understanding into the endemicity of this disease.

**Response to 1.4:**

We agree with the Reviewer on the importance of variability across ecological and sociological contexts and we have edited the manuscript to provide a more nuanced discussion of our findings, in particular, emphasising that MDA may not be appropriate in all contexts to tackle low-level transmission.

For example, where onchocerciasis and loiasis are co-endemic (principally in forested areas of Central Africa where *Chrysops* tabanid flies and *Simulium* blackflies co-occur), MDA may be inappropriate owing to the risk of severe adverse events (SAEs) following treatment with ivermectin of individuals with high *L. loa* microfilaraemia. The WHO Onchocerciasis Technical Subgroup (OTS), in its 8^th^ meeting (2024, unpublished) recommended integrating *L. loa* mapping with onchocerciasis elimination mapping (OEM) to assess the distribution of co-endemicity in currently untreated areas, with a Test and Not Treat (TaNT) strategy employed where loiasis is meso- or hyperendemic. Where loiasis is hypoendemic, MDA may be appropriate, albeit with pharmacovigilance. We have also referred to additional factors, such as civil unrest, which may make it harder for MDA to be initiated, irrespective of epidemiology.

We have added text to Abstract, Author Summary and Discussion sections to nuance our conclusions on the importance of initiating treatment to tackle low-level transmission in hypoendemic settings. In the Discussion we have included:

“Some of these communities may have already received MDA in lymphatic filariasis-onchocerciasis co-endemic areas [11]. However, the issue of starting MDA in hitherto untreated hypoendemic onchocerciasis communities raises important issues when these communities are also co-endemic with loiasis (another filarial infection caused by *Loa loa* and transmitted by *Chrysops* tabanid flies). This is because there is an increased risk of individuals with high *L. loa* mf loads (>20,000 mf/mL of blood) experiencing severe adverse events if treated with ivermectin [12,45]. Foci with moderate to high levels of loiasis endemicity would not be suitable for MDA. Here, Test and Not Treat (TaNT) strategies using LoaScope technology will be required [45]. Ivermectin MDA may be appropriate in foci with low loiasis endemicity. In both cases, pharmacovigilance/monitoring will be required [46]. To address these issues, the WHO OTS has suggested incorporating *L. loa* mapping into OEM [47]. Other factors, such as civil unrest or active conflict, may also hinder initiation of MDA, irrespective of local epidemiology.”

The new references are:

Loa Expert Committee Meeting 2024. Meeting Report. August 28-30, 2024 - Paris, France. <https://www.cor-ntd.org/sites/default/files/content/document/Loa%20Loa%20Expert%20Committee%20Meeting%20Report_AUG2024_FINAL.pdf>.

World Health Organization. Report of the Eighth Meeting of the WHO Onchocerciasis Technical Advisory Subgroup, Hybrid meeting, 9−12 December 2024.

**1.5:** While the manuscript discusses the limitations of current diagnostic tools, it lacks concrete suggestions for integrating findings with Ov16 seroprevalence data or other metrics. The authors need to integrate results with potential field diagnostics, such as Ov16 seroprevalence or blackfly infection rates, to bridge the gap between modeling and surveillance.

**Response to 1.5:**

We agree with the Reviewer that, ultimately, incorporating serological and entomological surveillance measures into our modelling framework will be of utmost importance to inform policy and programmatic decision-making. The work presented here is a first step towards these goals, providing the underpinning of future work that will translate (microfilarial) infection prevalence into serological and entomological indices. Indeed, we are in the latter stages of extending EPIONCHO-IBM to model anti-Ov16 seroprevalence. This is a substantial standalone piece of work, since the relationship between infection and anti-Ov16 serostatus has not previously been validated against paired infection-serology data. Therefore, in this manuscript we have focused on the stability of transmission in terms of the better understood dynamics of microfilarial prevalence. But in future, it is our goal to have a model that also integrates anti-Ov16 seroprevalence which will be essential for informing proposed serological thresholds for initiating MDA in areas undergoing onchocerciasis elimination mapping (OEM) and stopping decisions for verification of elimination. We are also starting work to model the prevalence of infected/infective blackflies. Again, this requires substantial model development to model the infection status of blackflies (e.g., using a compartmental structure). Currently, EPIONCHO-IBM models the mean number of L3 per blackfly which does not reflect entomological prevalence indices that are measured in the field and which are used for programmatic decisions (e.g., for stopping MDA and verifying elimination). Hence, in future publications, we intend to present an extended model—validated against entomological data—that will be more closely aligned with programmatic entomological measures.

**1.6:** The manuscript does not sufficiently discuss the implications of assuming closed populations or the limitations in generalizing findings across diverse onchocerciasis-endemic regions. The authors also need to elaborate on the operational challenges and recommendations for implementing the study’s insights in low-resource settings.

**Response to 1.6:**

We agree with the Reviewer that our assumption of a closed population is appropriately acknowledged and that the implications of this assumption are discussed further. We have expanded our discussion of this assumption in the revised manuscript, explaining that importation of infection could operate together with the density-dependent processes that are assumed to determine stability in a closed population (see **Response to 1.3**). We have also included further discussion on the challenges and resource implications of implementing MDA in an expanded geographical, ecological and sociological context, such as in onchocerciasis-loiasis co-endemic areas (see **Response to 1.4**).

**1.7:** The authors need to simplify jargon in the discussion. As it is, the discussion is too technical for a broader audience, that could include policymakers and field practitioners.

**Response to 1.7:**

We thank the Reviewer for helping us to make the manuscript as accessible as possible to a diverse audience. We have carefully reviewed the entire manuscript for jargon, either removing it or providing clarification where necessary.

**Reviewer #2:**

This paper provides an in-depth exploration of the stochastic fade out in a well-established onchocerciasis transmission model. The simulations, statistical analysis and figures are very clear, and it is well written. The main result, that hypo-endemic transmission areas should not be missed by treatment campaigns, has important policy implications. My only comments are relatively minor:

We thank the Reviewer for these highly appreciative comments.

**2.1:** I am not completely sold on the terminology. Borrowing the concept of thresholds from deterministic models and applying it to a stochastic model has its problems. This is rightly acknowledged in the limitations, there are no sharp thresholds. As such it is not clear why it is a useful concept, or at least, the manuscript doesn’t explicitly say why a threshold range is a useful concept. I think to strengthen the argument for defining it in this way, there should be an explicit description of how the system behaves (roughly) below, within, and above this range.

**Response to 2.1:**

We thank the Reviewer for raising this important point. We acknowledge that there is mathematical ambiguity in use of ‘threshold’ in the context of stochastic models (see Hardwick *et al*. [19] for further discussion). For example, unlike in a deterministic model, there is no strict threshold biting rate (TBR) below which transmission would not occur. In a stochastic sense, any biting rate will be associated with a probability of endemic transmission which will tend to zero for very low biting rates and to unity for high biting rates. However, as indicated by Reviewer 1, there is a need for a degree of pragmatism so that modellers can better interact and communicate with policy-makers. The wider onchocerciasis community is very focused on the use of ‘thresholds’, particularly for decision-making on when to start and stop MDA. This is why we defined a ‘threshold biting rate (TBR) range’ as being the range of annual biting rates for which the proportion of model runs with finite mf prevalence is >0% and <100%. That is, we explore the range of ABRs that give non-negligible (>0%) but less than ‘certain’ (<100%) probabilities of endemic stability (out of 300 repeat simulations, over a 500-year simulation timeframe, i.e., ≥1/300 and ≤299/300 chance of persistence). We believe that this approach maintains the terminology (‘threshold biting rates’) that is familiar to the wider onchocerciasis community, while also emphasising the probabilistic nature of persistence at low biting rates (ABRs) within this range.

We have clarified our use of the ‘threshold’ terminology in the revised manuscript, making it more explicit that outcomes (persistence or extinction) within the TBR range are subject to stochastic uncertainty.

We have added the following text to the Methods section under the **Threshold biting rates** subsection:

“We defined a TBR range as the ABRs for which a proportion (>0% and <100%) of 300 repeat model runs remained above 0% mf prevalence (measured across the total population) after the 500-year simulation period. Model runs generated by an ABR above the TBR range would ‘always’ persist (strictly, with probability >299/300) whereas below the TBR range, they would ‘never’ persist (strictly, with probability <1/300). When the ABR is within the TBR range, model runs persist with a non-degenerate probability distribution (i.e., with probability values between ‘always persisting’ and ‘never persisting’).”

**2.2:** There should be some mention about the reason why the observed decay rate is slow, much slower than other helminthic diseases. I.e. that the assumed fertile lifespan of the adult female parasite is very long. This means that it may be more important to treat these hypo-endemic areas than for other helminths with shorter lifespans, where perhaps hypo-endemic local R0 < 1 transmission is more likely to be sustained by importations from higher transmission areas.

**Response to 2.2:**

Thank you for raising this excellent point. We agree with the Reviewer that the long lifespan of female adult worms (up to a maximum of approximately 20 years according to our modelled Weibull distribution of adult worms’ survival times) leads to protracted transmission dynamics compared to other helminth infections with shorter lifespans. We have added the following text to include this important point in the Discussion:

“The assumed operation of these processes also enables modelling of very low mf prevalence, which are stable over epidemiologically-relevant timescales without importation of infection. The long lifespan of adult *O. volvulus* (we assume a maximum lifespan of 20 years with an average of 9-11 years [17,35]) facilitates protracted persistence which will be longer than for other helminths with substantially shorter lifespans (see [19] for an example in shorter lived helminths).”

Reference:

Plaisier AP, Van Oortmarssen GJ, Remme J, Habbema JDH. The reproductive lifespan of *Onchocerca volvulus* in West African savanna. Acta Trop. 1991; 48: 271–84.

**2.3:** In the supplementary, the Fig A caption mentions a breakpoint, which normally refers to the unstable equilibrium for R0 > 1 created by positive density dependent mating. For consistency this should be changed to threshold.

**Response to 2.3:**

We agree with the Reviewer and have updated the figure legend accordingly:

**“Figure A. Schematic representation of deterministic versus stochastic projections for low infection prevalence.** When run the model from a pre-established equilibrium using a fixed parameter set below the threshold for endemic persistence; a deterministic model predicts a monotonic decay in infection, since the annual biting rate (ABR) is below the transmission threshold (i.e., *R*_0_ is <1) and infection cannot persist.”

**Reviewer #3:**

Notes on Plos CompBio paper Stapley et al.

This is a well-written and interesting study using an existing stochastic model of onchocerciasis transmission to examine the stability of transmission in hypoendemic settings.

We thank the Reviewer for their positive comments on our work.

**3.1:** The results presented here challenge assumptions regarding transmission in hypoendemic settings, which raises important questions for onchocerciasis control policy guidelines if elimination of transmission is to be achieved. This points towards a need to dramatically review and expand the current targeting of elimination efforts.

**Response to 3.1:**

We thank the Reviewer for highlighting the important implications of our work.

**3.2:** Whilst there is clear value in studies that challenge existing policies and the assumptions that underly them, it is a little disappointing given the operational implications for this work that there is no clear evidence of engaging with stakeholders. It would really strengthen the authors’ position if they could demonstrate stakeholder engagement, either as co-authors or formally acknowledged through, for example, a PRIME-NTD table, and I would anticipate input from stakeholders to help ground both the scenarios modelled and the conclusions within the wider context of onchocerciasis control efforts.

**Response to 3.2:**

We completely agree with the Reviewer. We erroneously omitted a PRIME-NTD table from the original submission (now referred to in the Main Text and included in the Supporting Information file) which describes how engagement with stakeholders from the World Health Organization, Gates Foundation, Task Force for Global Health and Centers for Disease Control and Prevention was crucial in motivating this study. We refer to this stakeholder engagement in the PRIME-NTD table (S1 File). We have added the following to the Methods:

“We adhered to the five principles of the Neglected Tropical Diseases (NTD) Modelling Consortium regarding Policy-Relevant Items for Reporting Models in Epidemiology of NTDs (PRIME-NTD), for good practice in NTD modelling [25] (S1 File Text B and Table B)."

Reference:

Behrend MR, Basáñez MG, Hamley JI, Porco TC, Stolk WA, Walker M, de Vlas SJ, NTD Modelling Consortium. Modelling for policy: the five principles of the Neglected Tropical Diseases Modelling Consortium. PLoS Negl Trop Dis. 2020; 14: e0008033.

Other points:

**3.3:** Why are 500 year simulations used? Why 300 model runs? 500 years doesn’t seem to be an epidemiologically relevant timescale, and it would be more interesting to see a shorter timescale examined in more detail.

**Response to 3.3:**

We thank the Reviewer for providing us the opportunity to further clarify these details. Simulations lasting 500 years were used as a methodological approach to generate model outputs over a long time-horizon so that the dynamics of persistence and stochastic fade-out could be fully evaluated. This time horizon is not intended to be epidemiologically relevant to policymakers. This is why, in Figure 3, we show the evolution of mf prevalence across a 150-year period, in increments of 10-years to provide a more relevant scale, whilst capturing longitudinal dynamics.

Three hundred repeat simulations for each combination of parameters were used as it is sufficient to capture the stochastic variation generated by the model (e.g., we can estimate persistence even at probabilities as low as 1/300, see **Response to 2.1**), also within computationally practical limits.

We have added text to the Methods section, under the **Initial conditions** sub-section to further clarify these points:

“Once this equilibrium was established, we set time to *t* = 0 days and dropped the ABRs to the TBR range under consideration (informed by [17]), running simulations from *t* = 0 days for 500 years, repeated 300 times. A 500-year simulation period was chosen to be an arbitrary long timeframe to evaluate comprehensively the dynamics of persistence and stochastic fade out over time, whilst remaining within computationally accessible limits. Similarly, the 300 repeats were chosen to ensure that persistence could be detected even at an arbitrarily low probability (1/300) and provide a near-continuous scale on the relationship between persistence probability and ABR”.

**3.4:** What informed the values of k_E_ used (0.2-0.4)? Were these derived from fitting to data?

**Response to 3.4**

These values were motivated from fitting the EPIONCHO-IBM model to microfilarial prevalence versus ABR data from a range of transmission settings across Africa, as done in Hamley *et al.* (2019).

We have added text to the Methods section under the **Threshold biting rates** subsection to clarify this:

“and the magnitudes of inter-individual exposure heterogeneity from *k_E_* = 0.2 to *k_E_* = 0.4, using corresponding density dependence parameters, with values informed by previous model fitting to paired parasitological and entomological data [17] (S1 File, Table A).”

**3.5:** Given the impact of population size on stochastic processes/fade out, why were population sizes of N=50-1000 used? Was this informed by demographic data from endemic areas?

**Response to 3.5:**

This is another important clarification raised by the Reviewer. These population sizes (*N* = 50-1000) are typical of villages across onchocerciasis transmission settings in Africa as documented in Katabarwa *et al.* (2010) and O’Hanlon *et al.* (2016).

We have added text to the Methods section under the **Threshold biting rates** subsection to clarify this:

“This was done by varying the human population size *N* from 50 to 1000 (values typical of endemic villages across SSA [13,22])”

**3.6:** How could k_E_ and/or ABR be expected to change under interventions? It would be interesting to see simulations with more varying levels of heterogeneity, and given the challenges of MDA in some hypoendemic settings due to co-endemic loiasis, it would be interesting to see simulations of interventions targeting the vector, which may reduce ABR/impact heterogeneity.

**Response to 3.6:**

The focus of this paper is on evaluating the stability of transmission in the absence of intervention, with our findings challenging previous assertions that transmission in hypoendemic settings is likely to fade out, in epidemiological relevant timescales, by implementing control interventions in neighbouring meso- or hyperendemic foci. Therefore, it is beyond the scope of the manuscript to evaluate how model parameters may change under interventions. However, an important limitation of the current work, which we highlight in the revised manuscript, is that we assume that the annual biting rate (ABR) of blackfly vectors remains constant over time. While this is reasonable because we are not considering vector control interventions—which would certainly reduce ABR—it will not capture secular changes in ABR, related to climate change or other anthropogenic effects (e.g., deforestation, pollution of blackfly breeding sites). Furthermore, it has been hypothesised that there is a likely relationship between ABR and *k_E_*, by which *k_E_* would be expected to increase (decreasing exposure heterogeneity) for increasing ABR values. Indeed, previous work (Hamley *et al.,* 2019) has shown that at high ABRs—consistent with hyper- and holendemicity—EPIONCHO-IBM captures better the ABR-microfilarial prevalence relationship using higher values of *k_E_* (*k_E_*=0.4), and vice versa (*k_E_*=0.2 capturing better lower prevalence at low ABRs. However, unpublished modelling work fitting EPIONCHO-IBM to OCP village-level parasitological data has only found a modest relationship between these two parameters.

We agree with the Reviewer on the challenges associated with implementing MDA in onchocerciasis-loiasis co-endemic foci and our discussion of this has been expanded in the revised version of the manuscript (see **Response to 1.4**). We also agree with the Reviewer that since the findings of this work indicate the expansion of interventions to low-transmission onchocerciasis settings, a natural next step and follow up manuscript should explore the impact of MDA in this context to evaluate timeframes to elimination under different options.
